# Metal-organic framework and Tenax-TA as optimal sorbent mixture for concurrent GC-MS analysis of C1 to C5 carbonyl compounds

**DOI:** 10.1038/s41598-018-23391-6

**Published:** 2018-03-22

**Authors:** Tanushree Dutta, Ki-Hyun Kim, Richard J. C. Brown, Yong-Hyun Kim, Danil Boukhvalov

**Affiliations:** 1 Department of Civil and Environmental Engineering, Hanyang University, 222 Wangsimni-Ro, Seoul, 04763 Korea; 2 Department of Chemical, Medical and Environmental Science, National Physical Laboratory, Teddington, TW11 0LW UK; 3 Jeonbuk Department of Inhalation Research, Korea Institute of Toxicology, Jeongeup, 56212 Republic of Korea; 4 Department of Chemistry, Hanyang University, 222 Wangsimni-Ro, Seoul, 04763 Korea

## Abstract

We report a multi adsorbent-based method using combinations of metal-organic frameworks (MOFs) and a commercial sorbent Tenax-TA for sampling and thermal desorption (TD) gas chromatography-mass spectrometry (GC-MS) quantification of mixtures of six (C1 to C5) aldehydes. The feasibility of this approach was demonstrated along with the optical analytical conditions for maximum recovery. Optimal TD conditions for adsorption and desorption of aldehydes using MOF-5 (Zn-based MOF)+ Tenax-TA were determined as −25 °C and 150 °C, respectively (purge volume: 100 ml). These conditions yielded good linearity (R^2^ = 0.997), precision, and high sensitivity. Analysis of the aldehyde mixtures yielded slightly smaller R^2^ values than the analysis of single species. Additionally, the performance of MOF-5+ Tenax-TA was compared with other combinations comprising of Cu-based MOF-199 and Zr-based MOF of UiO-66 topology. The results of the theoretical modelling analyses propose simultaneous interaction of the C=O group of aldehydes with open metal sites of the studied MOFs and van der Waals interaction of hydrocarbon “tail” of aldehydes with linkers of MOFs. The combined interactions significantly increased the enthalpy (eV/molecule) of formaldehyde adsorption on MOF. Our findings unravel a potential way to extend the application of GC-based detection toward concurrent analysis of organic molecules of variable sizes.

## Introduction

Metal organic frameworks (MOFs) are hybrid nanoporous materials comprised of metal ion or metal ion clusters and bridging organic linkers. Remarkable progress has been made in the application of MOFs in the fields of gas storage, catalysis, sensing, gas adsorption, and separation of chemicals due to their large surface area, tunable pore structure, and reasonably high thermal, chemical, and mechanical stability^1,2^. MOFs are also recognized as effective sorbents for the pre-concentration, extraction, and detection of trace analytes^3^. The potential of MOFs as a sorbent material for sampling/trapping and as a stationary phase for chromatographic separation of alkane isomers, branched alkanes, benzene homologues, and alkyl-aromatics has been well established^4–11^.

Thermal desorption (TD)-gas chromatography (GC) is one of the most important tools for the quantitative analysis of volatile organic compounds (VOCs). This analysis can be effectively carried out for light VOCs like carbonyl compounds (CCs) using a high performance liquid chromatography (HPLC)-UV system in the context of a derivatization technique. The derivatization step is particularly critical for the quantification of gaseous formaldehyde (FA). Note that it is impractical to quantify gaseous FA using commercial sorbents like the Carbopack series, Carboxen, or Tenax-TA due to their poor specific adsorptivity for FA, resulting in short breakthrough times^12,13^. The limitations of the poor adsorptivity of commercial adsorbents can be overcome by replacing the commercial sorbents or by combining them with materials with large surface area and high thermal stability. MOFs are nanoporous materials with a larger surface area than commercial adsorbents (surface area (m^2^·g^−1^): (1) MOF-5 = 2,205, (2) MOF-199 = 1,264, (3) UiO-66 = 1,580, (4) Carboxen 1000 (60/80 mesh) = 1,200, (5) Carbopack X (60/80 mesh) = 240, and (6) Tenax TA (60/80 mesh) = 35)^14–16^. Apart from their unique surface characteristics, MOFs are reasonably stable compounds with a robust crystalline structure.

Carbonyl compounds (CCs), which consist of a carbon atom double bonded to an oxygen atom (C=O), are ubiquitous in the troposphere and play a significant role in secondary photochemical reactions^17^. Formaldehyde (FA) and acetaldehyde (AA) are classified as toxic air pollutants due to their adverse effects on public health and the environment^18^. Carbonyl compounds (except FA) are most commonly analyzed by trapping the compounds onto commercial sorbents and transferring them into a thermal desorption unit connected to a GC or GC-MS system^19^. FA, on the other hand, cannot be quantified by trapping on commonly available sorbent materials. Analysis of FA generally requires complex derivatization for stable sampling and accurate analysis for any kind of chromatographic system (e.g., GC or LC). Derivatization using the 2,4-dinitrophenylhydrazine (DNPH) cartridge method is frequently used to convert low molecular weight FA to a high molecular weight complex for analysis using HPLC-UV methods^20^. Likewise, mixtures of CCs containing FA cannot be quantified using a TD-GC/MS system due to the same sorptive limitations of commercial sorbents.

The reliability of MOF-5 (organic linker: 1,4-benzenedicarboxylates and metal cluster: Zn_4_O_13_) as a sorptive media for the collection of FA (and the subsequent analysis by TD-GC/MS) has been demonstrated previously^21,22^. Nonetheless, the use of such media for simultaneous quantification of low and high molecular weight C1 to C5 aldehydes has not been investigated. In this work, we developed a multi-adsorbent TD–GC/MS method to analyze a mixture of environmentally important carbonyl species, i.e., formaldehyde (FA), acetaldehyde (AA), propanaldehyde (PA), butaraldehyde (BA), isovaleraldehyde (IA), and valeraldehyde (VA). A new sorption method was developed to absorb both light and heavier carbonyls simultaneously by combining MOF-5 and a well-known commercial sorbent, Tenax-TA. Mixtures of CCs were analyzed using sorbent tubes (ST) and cold traps (CT) packed with MOF and Tenax-TA. The MOFs for this study were chosen based on their high thermal, mechanical, and chemical stability: zinc (Zn)-based MOF-5, copper (Cu)-based MOF-199, and zirconium (Zr)-based UiO-66 (amine derivative). All three MOFs are stable (250 to 300 °C) at the operating temperatures in TD-GC/MS. MOF-199, which contains both copper (Cu^2+^) ions and open metal sites, exhibits greater chemical stability than MOF-5, which contains zinc (Zn^2+^) tetrahedrons. Likewise, although zirconium (Zr)-based MOFs (of UiO-66 topology) are thermally less stable than Zn-based MOF (MOF-5), they exhibit greater mechanical stability than MOF-5 and MOF-199. The reliability of the analytical system was evaluated by measuring basic quality (QA) assurance and quality control parameters. Furthermore, the outcomes of our experiments were discussed in light of theoretical interactions between MOFs and the main active site of aldehyde (C=O group) molecules based on density functional theory (DFT). As such, this study reveals for the first time the use of MOF-based multi-adsorbents in sorbent tubes (at room temperature) and thermal desorption-based analysis of a mixture of carbonyl compounds with varying molecular weights.

## Results and Discussion

### **C**haracterization of synthesized MOFs

The morphology, degree of crystallinity, thermal stability, and chemical functionalities of synthesized MOFs were determined through PXRD, SEM, FTIR, and TGA analyses. The obtained PXRD data of MOF-5 (see Supplementary Fig. S1) were consistent with the previously published results (See Gu *et al*.^21^). The relative peak intensities of activated MOF-5 showed characteristic diffraction peaks at 2Ѳ values of 6.7° and 9.6°. Likewise, the PXRD patterns of UiO-66-NH_2_ (diffraction peaks at 2Ѳ of 7.2° and 8.3°) (see Supplementary Fig. S2) and MOF-199 (diffraction peaks at 2Ѳ of 11.6°, 13.4°, 14.6°, 16.4°, 17.4°, 19.0°, and 29.4°) (see Supplementary Fig. S3) matched well with the literature^23–25^. The FE-SEM morphologies of the synthesized MOFs confirmed the formation of crystals with particle sizes ranging from 150 to 300 nm.

The two sharp FTIR bands of MOF-5 located in the region of 1650–1300 cm^−1^ were attributed to the attachment of the carboxylate ligand to the Zn_4_O cluster^26^. The FTIR bands of pristine and used MOF-5 were consistently demonstrating minimum disruption of metal-ligand binding by the adsorption and subsequent desorption of carbonyls (Supplementary Fig. S4). The peaks centered at 3500 cm^−1^ indicated OH groups involved in H-bonding^27^. FTIR peaks in the region of 3100–2900 cm^−1^ indicated the aromatic and aliphatic ν(CH) stretching vibration of benzene rings and DMF. The relatively shorter bands located between 800–1200 cm^−1^ indicated Zn-O stretching. FTIR peaks of the MOF-199 and the amine derivative of UiO-66 were in good agreement with previously published results. MOF-199 showed two peaks at 1108 and 760 cm^−1^, indicating C-O-Cu stretching vibrations^28^. The symmetric and asymmetric stretching vibrations of the carboxylate groups were observed at 1554, 1447, and 1367 cm^−1^. UiO-66(NH_2_) showed characteristic bands at 1257 and 1383 cm^−1^, representing C-N stretching^29^. IR peaks at 765 and 660 cm^−1^ were attributed to N-H vibrations.

The TGA of MOF-5 was in excellent agreement with the previously reported results of Gu *et al*.^21^. The TGA results showed an initial weight loss of 12% from 100 to 300 °C, representing the loss of DMF molecules in the MOF pore space. This was followed by another weight loss of 35% from 300 to 500 °C due to the breakdown of the MOF structure. Thus, the activated MOF-5 was found to be thermally stable at the operation temperatures (<200 °C) used in this study. Similarly, UiO-66(NH_2_) and MOF-199 showed good thermal stability (demonstrated by less than 15% loss of weight due to removal of solvent molecules) below 200 °C.

### Quantification of a mixture of CCs (including FA) using multi-adsorbent-based TD-GC/MS

**Figure 1:**
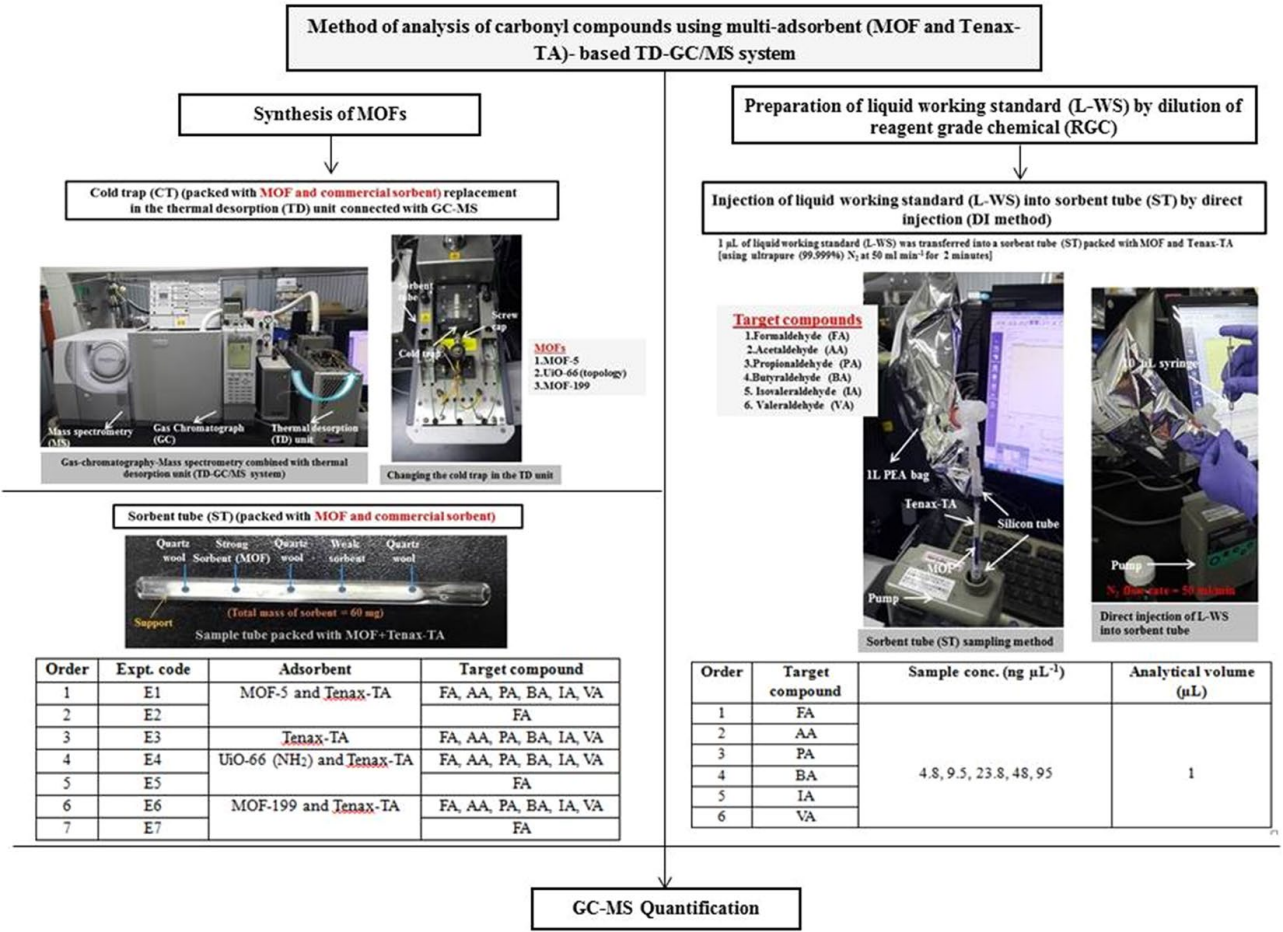
Pictorial representation of the method of analysis of carbonyl compounds using a multi-adsorbent (MOF and Tenax-TA)-based ST-TD-GC/MS system. Left panel (A) shows the method of replacement of the cold (quartz) trap in the TD unit interfaced with GC/MS system and the right panel and sorbent tube (ST), and (**B**) demonstrates the injection of L-WS into the sorbent tube (ST) by direct injection (DI).

In this study, L-WS of FA (on its own) and a mixture of 6 CCs (including FA as a component of the mixture) (Table 1) were analyzed using a multi-adsorbent (MOF and Tenax-TA)-based TD-GC/MS system (Table 2). The use of MOF-5 for the analysis of FA was investigated previously by Gu *et al*.^21^ and Kim *et al*.^22^. Gu *et al*.^21^ developed an in-field sampling and pre-concentration technique for the TD-GC/MS determination of atmospheric FA without requiring chemical derivatization of the FA molecules^22^. Kim *et al.*^22^ determined the optimal analytical conditions for maximum FA recovery. However, the application of MOFs to the analysis of a mixture of CCs has not been reported previously. This could be because high molecular weight carbonyls like AA, PA, BA, IA, and VA can be easily quantified using various commercially available sorbents. However, in the presence of FA, the commercial sorbents become ineffective, requiring more advanced techniques. Combining commercial sorbents with MOFs (e.g., MOF-5), which has strong adsorptivity for FA, overcame the sorptive limitations of commercial sorbents at very low molecular weights (Refer to Fig. 1 and Supplementary Fig. S5 for the experimental scheme). Thus, the combined use of MOFs with commercial sorbent enabled simultaneous quantification of low and high molecular weight CCs in the TD-GC/MS system. All six CCs were detected simultaneously using the MOF-based multi-adsorbent technique. To the best of our knowledge, this is the first study that demonstrated the use of MOFs for the quantification of mixtures of CCs (including FA).

**Table 1:** Characteristics of the target compounds.

Order	Full name	Short name	MW (g/mol)	Density (g mL^−1^)	Formula	CAS number	Extracted Ion chromatogram	Carbon number
1	Formaldehyde	FA	30.03	0.8153	CH_2_O	50-00-0	29	1
2	Acetaldehyde	AA	44.05	0.7850	C_2_H_4_O	70-07-0	44	2
3	Propionaldehyde	PA	58.1	0.8100	C_3_H_6_O	123-38-6	58, 59	3
4	n-Butyraldehyde	BA	72.1	0.8050	C_4_H_8_O	123-72-8	41–44, 72	4
5	Isovaleraldehyde	IA	86.1	0.7970	C_5_H_10_O	590-86-3	41–44	5
6	n-Valeraldehyde	VA	86.1	0.8100	C_5_H_10_O	110-62-3	57	5

**Table 2:** The experimental scheme used to develop the ST-TD-GC/MS method using MOF and Tenax-TA for the analysis of carbonyl compounds.

Experimental stages					
Order	Adsorbent	Experimental Code	Description		
1	**MOF-5 + Tenax-TA**	**E1**	**Optimization of the ST-TD-GC/MS system using MOF-5 + Tenax-TA as a sorbent**		
			Adsorption temperature of the cold trap in the TD system		
			Desorption temperature of the ST and the CT		
			Pre-purge condition of the liquid FA standard		
			**Calibration & QA/QC analysis using the optimal ST-TD-GC/MS conditions**		
2		**E2**	**Analysis of FA at optimal (from E1) TD-GC/MS conditions**		
3	**Tenax-TA**	**E3**	**Analysis of a mixture of carbonyl compounds**		
4	**UiO-66(NH**_**2**_ **) + Tenax-TA**	**E4**	**Analysis of a mixture of carbonyl compounds**		
5		**E5**	**Analysis of FA**		
6	**MOF-199 + Tenax-TA**	**E6**	**Analysis of a mixture of carbonyl compounds**		
7		**E7**	**Analysis of FA**		
**Details of E1**					
**Order**	**Expt. code**	**Adsorption temp. of the CT (°C)**	**Desorption temp. of the ST and CT (°C)**	**Pre-purge volume (mL)**	**Standard phase**
1–1	E1–1	−25	150	100	Liquid
1–2	E1–2	−25	150	250	Liquid
1–3	E1–3	−10	150	100	Liquid
1–4	E1–4	−10	150	250	Liquid
1–5	E1–5	−10	100	100	Liquid
1–6	E1–6	−10	100	250	Liquid
1–7	E1–7	−25	100	100	Liquid
1–8	E1–8	−25	100	250	Liquid

### Optimum analytical conditions for recovery of mixtures of carbonyls

Factors influencing the analysis of individual CCs (FA, AA, PA, BA, IA, VA) in a mixture were examined using a system with MOF-5 and Tenax-TA-based sorbent tube (ST) sampling, a pre-concentration step, and a TD-GC/MS (Table 3). We collected a mixture of FA, AA, PA, BA, VA, and IA in a ST packed with MOF-5 and Tenax-TA and analyzed the concentration of each compound in a TD-GC/MS system. The quartz trap (of the thermal desorption system) packed with MOF-5 and Tenax-TA was used for subsequent adsorption and desorption of CCs before analysis in the GC/MS system. The optimal analytical conditions for the recovery of carbonyls were determined by comparing the recovery of the CCs at different CT and ST adsorption and desorption temperatures and at different ST pre-purge conditions. The results of the analysis of CCs are summarized in Table 4.

**Table 3:** Optimal conditions for the analysis of carbonyls using TD-GC/MS.

Thermal desorber (model: UNITYII, Markes, UK)			
***Sampling tube***			
1. Trap tube	Quartz (length: 90 mm, OD: 6.4 mm, and ID: 4.2 mm)		
2. Adsorbent	MOF-5 + Tenax TA		
3. Desorption time	5 min		
4. Desorption flow	10 mL min^−1^ (to cold-trap)		
5. Desorption temp.	150 °C		
***Cold-trap***			
6. Trap tube	Quartz (length: 100 mm, OD: 3.2 mm, and ID: 2 mm)		
7. Adsorbent	MOF5 + Tenax-TA in quartz trap		
8. Adsorption temp.	−25 °C (from sampling tube)		
9. Desorption temp.	150 °C (to GC)		
10. Desorption flow	10 mL min^−1^		
***Carrier gas setting***			
11. Carrier gas	Helium (>99.999%)		
12. Split flow	10 mL min^−1^		
*Line and interface Temp*.*: 150* *°C*			
**Gas chromatography** (**model: GC-2010**, **Shimadzu**, **Japan**)			
13. Column	CP-wax (Agilent J&W, USA)		
	(length: 60 m, diameter: 0.25 mm, and film thickness: 0.25 µm)		
14. Oven setting	40 °C (5 min) → 24 °C/min → 220 °C (5.5 min)		
	(Total program time = 18 min)		
**Mass spectrometry** (**model: GCMS-QP2010 ultra**, **Shimadzu**, **Japan**)			
15. Ionization mode	EI (70 eV)	18. SIM mode (0.5 to 5 min)	29, 30, and 44
16. Ion source temp.	230 °C	19. TIC scan range (5 to18 min)	35–600 m/z
17. Interface temp.	230 °C	20. Scan speed	1250

**Table 4:** Comparison of analysis results of the mixture of aldehydes using MOF-5 + Tenax-TA as ST and CT sorbents.

Order	Compound	Adsorption temp. = −25 °C								Adsorption temp. = −10 °C							
		Desorption temp = 150 °C				Desorption temp = 100 °C				Desorption temp = 150 °C				Desorption temp = 100 °C			
		Purge volume = 100 ml		Purge volume = 250 ml		Purge volume = 100 ml		Purge volume = 250 ml		Purge volume = 100 ml		Purge volume = 250 ml		Purge volume = 100 ml		Purge volume = 250 ml	
		RF value	R^2^	RF value	R^2^	RF value	R^2^	RF value	R^2^	RF value	R^2^	RF value	R^2^	RF value	R^2^	RF value	R^2^
1	FA	2,762	0.8086	1,342	0.9599	1,081	0.7968	1,508	0.7136	2,152	0.9959	1,396	0.8938	766	0.7509	831	0.766
2	AA	—	—	—	—	—	—	—	—	2,117	0.9829	777	0.9953	—	—	—	—
3	PA	3,967	0.9902	3,775	0.9901	3,612	0.9935	3,330	0.9883	3,363	0.9723	3,000	0.9858	3,079	0.9961	2,770	0.993
4	BA	10,879	0.9936	13,207	0.9940	11,165	0.9972	11,806	0.9963	13,372	0.9958	13,757	0.999	10,696	0.9933	11,578	0.997
5	IA	14,190	0.9768	18,637	0.9875	17,651	0.9841	15,478	0.9772	19,075	0.9837	19,954	0.9908	13,112	0.9853	14,939	0.983
6	VA	13,097	0.9768	16,304	0.9802	15,637	0.9868	17,022	0.9881	18,674	0.9847	16,978	0.9892	13,706	0.9810	13,933	0.983

Optimal TD conditions for the analysis of FA (in mixture of CCs) were: (i) an adsorption temperature of CT of −25 °C, (ii) desorption temperatures of CT and ST of 100 °C, and (iii) a pre-purge volume of 100 mL. The results indicated that the combined effects of several key factors (e.g., adsorption temperature, desorption temperature, and pre-purge volume) were critical to determine the analytical conditions for a mixture of CCs. For instance, the RF values of FA increased with decreasing adsorption temperature when the ST and CT were desorbed at 150 °C (ST pre-purge volume = 100 ml). The recovery of PA decreased with increasing adsorption temperature. This trend was reversed for BA, IA, and VA when a desorption temperature of 150 °C was used. The RF values of FA were slightly greater at ST and CT desorption temperatures of 150 °C, with one exception. The R^2^ values for the FA calibrations in a CC mixture were smaller than the R^2^ values of FA alone, which clearly indicates that the recovery of FA decreased in the presence of high MW compounds. The adsorption stability and solvent effect were assessed using different pre-purge volumes of L-WS of CCs. The FA peak was clearly distinguishable from the methanol (solvent), similar to the previous findings of Kim *et al*.^22^. No effect of pre-purge volume on CC recovery was found in this study. It is worth noting that previous FA analysis study using MOF-5 by Kim *et al*.^22^ suggested a possible breakthrough of FA before the sampling volume reached 250 ml.

The basic calibration and QA data were obtained by analyzing the L-WS of the mixture of CCs using the optimal TD conditions used for the analysis of FA (in mixture of CCs), as mentioned above (Table 5). The method detection limit (MDL) for the CC analysis was determined using a three-fold dilution of the first calibration point (i.e., 4.8 ng/µL) with methanol to reach a final concentration of 1.6 ng/µL (injection volume = 1 µL). The MDL was calculated as the product of the standard deviation of seven replicates and the Student’s t-value at the 99% confidence level (for example: 6 df, t = 3.14). The MDL of FA in the CC mixture with high MW compounds (MDL ≥ 1 ng in this study) was greater than the analysis of FA alone (MDL (FA) = 0.1 ng^22^). The relative standard error (RSE) values (calculated by triplicate analyses of the third (23.8 ng) calibration point) ranged from 2 to 4% (except for AA).

**Table 5:** Calibration and quality assurance data for the analysis of carbonyl compounds (using MOF-5 + Tenax-TA as the sorbent media) by TD-GC/MS analysis.

Method detection limit (MDL)							
Order	Compound	Analytical mass (ng)	Mean	SD	MDL^a^		Conc.^b^
					Peak area	**Mass (ng)**	
1	FA	1.6	10,611	2,151	6,756	2.45	1.99
2	AA	1.6	18,205	7,414	23,281	—	—
3	PA	1.6	4,167	1,436	4,510	1.15	0.48
4	BA	1.6	26,375	3,451	10,835	1.01	0.34
5	IA	1.6	42,437	9,823	30,846	2.17	0.62
6	VA	1.6	48,416	10,403	32,666	2.49	0.71
**Relative standard error (RSE, %)**							
**Order**	**Compound**	**Analytical mass (ng)**	**Peak area (Unitless)**			**Mean (Unitless)**	**Relative standard error (RSE, %** ^**c**^ **)**
1	FA	23.8	49,168	44,380	43,408	45,652	3.90
2	AA	24.1	90,532	75,531	76,826	80,963	5.93
3	PA	23.9	94,571	89,391	86,817	90,260	2.53
4	BA	24.6	240,442	266,216	261,807	256,155	3.11
5	IA	23.9	383,396	418,616	411,292	404,435	2.65
6	VA	24.3	354,054	394,974	380,209	376,412	3.18

^a^MDL was calculated as the product of the standard deviation of seven replicate measurements using a 1 µL injection volume of mixture of CCs (1.6 ng μL^−1^) (prepared by the 3-fold dilution of the 1st calibration point of L-WS, i.e., 4.8 ng μL^−1^, with methanol) multiplied by the Student’s t-value at the 99.9% confidence level (6 df, t = 3.14). ^b^Calculated by assuming a total sample volume of 1000 mL (25 °C). ^c^Calculated by the triplicate analyses of the third (23.8 ng) calibration point.

### Comparison of MOF-5 with other MOFs

The method of analyzing the CC mixture using MOF-5 combined with Tenax-TA as the ST and CT sorbent for TD-GC/MS was compared with other MOFs with good thermal and mechanical stability including: (i) a Cu-based MOF (MOF-199) and (ii) a Zr-based MOF (of UiO-66 topology). The MOFs were carefully selected based on the outcomes of previous studies. MOF-199 has been used previously for competitive adsorption experiments^14,30^. Also, MOF-199 is a rare example of MOFs that has been developed at an industrial scale^31^. The amine derivate of UiO-66 (i.e., UiO-66(NH_2_)) was chosen based on its previous use for FA sensing^29^. As in the previous analysis using an MOF-5 based system, the analyses using Cu- and Zr-based MOFs were carried out with L-WSs of both FA alone and a mixture of carbonyls including FA. The mass spectra of the target analytes for different MOF types (as shown in Supplementary Figs S6, S7, and S8) are plotted to confirm the retention time of each analyte. FA was detected using both the MOF-199 + Tenax-TA- and UiO-66-(NH_2_) + Tenax-TA-based sorbents. However, the performances of MOF-199 + Tenax-TA and UiO-66-(NH_2_) + Tenax-TA were poor compared to that of MOF-5 + Tenax-TA. Both MOF-199 + Tenax-TA- and UiO-66-(NH_2_) + Tenax-TA-based methods generated very low (sometimes negative) R^2^ values. The exceptionally low R^2^ values (linearity) might be due to the formation of derivatives of FA and AA (as confirmed by the appearance of additional peaks during analysis of CC mixtures (see Supplementary Fig. S7)).

### Mechanism and energetic of adsorption of single aldehyde species versus combination of aldehydes based on DFT calculations

Results of the calculations demonstrated that for all three studied hosts and for all types of aldehydes, formation of two types of chemical bonds occurred simultaneously. One is a coordination bond between the oxygen from C=O head of aldehydes and open metal sites of MOFs^29^. The second one is a van der Waals (vdW) interaction of the hydrocarbon “tail” of the aldehydes with linkers of MOFs. For all types of MOF, calculations demonstrate decrease of the enthalpy of adsorption with the increasing contribution of the hydrocarbon “tail” of aldehydes.

**Figure 2:**
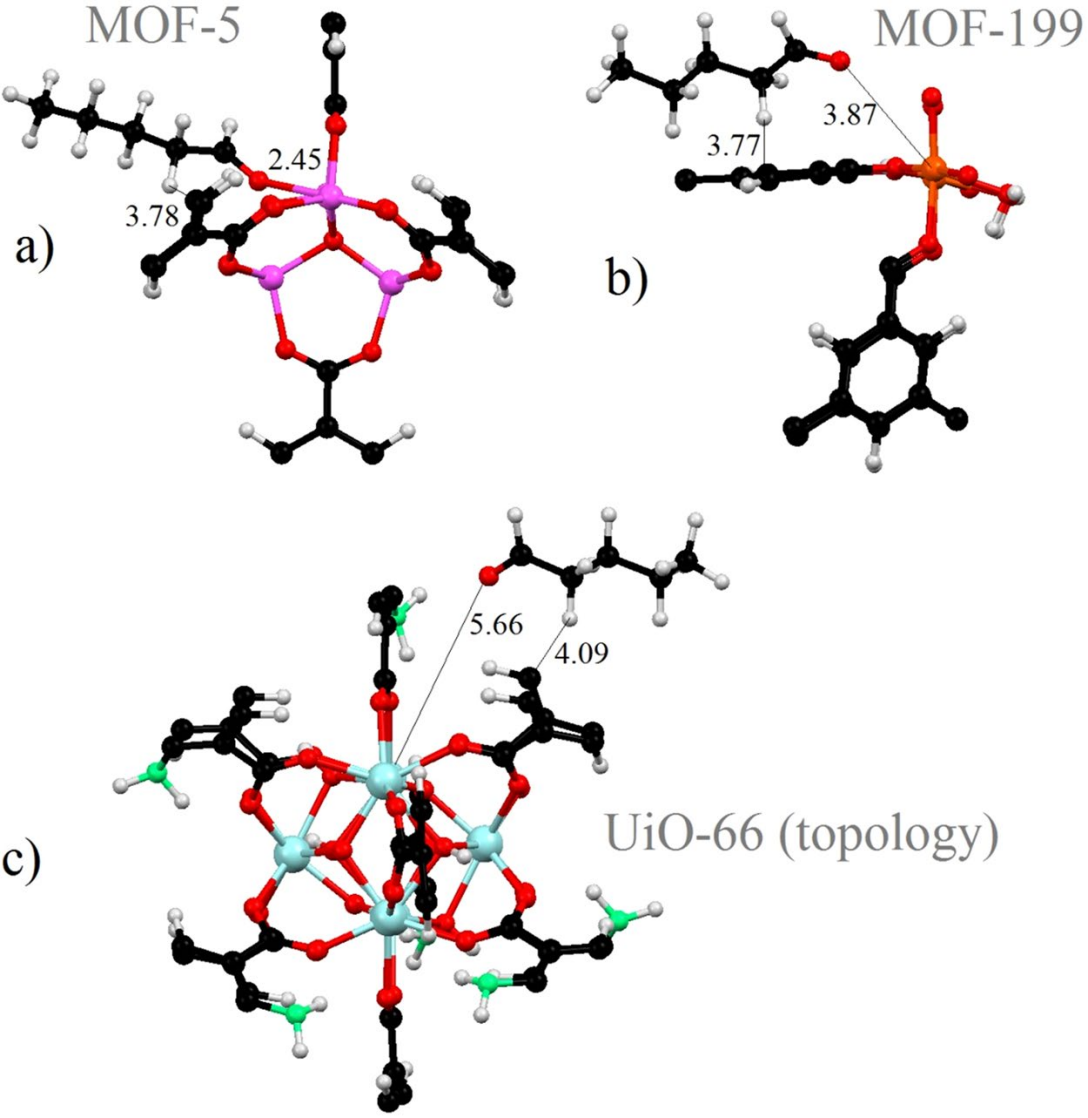
Optimized atomic structure of parts of the supercell of MOF-5 (**a**), MOF-199 (**b**) and UiO-66 MOF (**c**) with adsorbed VA. The numbers (in Å) is corresponding with the distances between oxygen and open metal sites of MOFs and distances between the “tail” of VA and linkers of MOFs.

Different geometry of metal sites and pores of studied MOFs provides different contribution from two above-described types of bonds to the energetics of adsorption (see Fig. 2). In case of MOF-5, the distance between oxygen atom of aldehydes and Zn-ion is in the order of 2.5 Å. This distance corresponds to the leading role of metal-oxygen coordination bonds in the interaction of the aldehydes with MOF-5. In contrast, in the cases of MOF-199 and especially UiO-66-NH_2_, the distances between open metal site and oxygen “head” of the aldehydes are visibly larger. Because coordination bonds are much more robust than vdW bonds, the magnitude of the enthalpy in adsorption of MOF-5 is significantly larger than those of the other two MOFs (see Supplementary Tables S1, S2 and S3).

On the other hand, the interaction between the hydrocarbon “tails” of aldehydes and the linkers of MOF-199 (or UiO-66-NH_2_) provides visible distortion of the lattice of these MOFs. To characterize these distortions, we calculated the ratio of the volumes of the supercells of pristine MOFs and MOFs after adsorption. In the case of MOF-5, the change of the calculated volume of MOF in result of the adsorption is within 2%, in contrast to much larger values for other MOFs (6% for MOF-199, and 8% for UiO-66-NH_2_). This result is in agreement with the previous calculations by Wu *et al*.^32^ in which the rigidity of MOF-5 and flexibility of UiO-66-NH_2_ (at low mechanical loading) were demonstrated. The magnitude of deviation of the volume after adsorption from the value for pristine systems increases with the increasing length of aldehydes i.e. with the increased number of vdW interactions between hydrocarbon “tail” and linkers. The observed distortion of the MOF increases the total energy of the system which makes adsorption less energetically favorable. Results of the calculations demonstrate that despite formation of additional vdW bonds between larger aldehydes and MOF-199 and UiO-66-NH_2_, the magnitude of the enthalpy of adsorption change insignificantly (see Supplementary Tables S2 and S3) in contrast to MOF-5 (see Supplementary Table S1).

The next step of our study was to assess how the enthalpy of adsorption and volume of MOF changed due to the adsorption of the second species of the same or another kind with an already adsorbed molecule of the first type. In case of adsorption of multiple species with larger sizes (PA, BA, VA, and IA) onto MOF-5, the values of the adsorption enthalpy and the local distortion of MOF were almost the same as for the cases of single species adsorption. However, in the presence of the paired carbonyl (eg., between FA and AA), a significant increase in the enthalpy of adsorption was observed for both species. This effect can be interpreted as the result of a mismatch in the local distortion of MOF caused by adsorption of small species which interacted only with the Zn-core of the MOF-5. As the enthalpy of adsorption remained largely negative, the total quantity of adsorbed aldehyde should be (almost) the same. Therefore, the results of this analysis suggest that larger molecules should be preferentially adsorbed over smaller molecules when a mixture of aldehyde is present. In case of adsorption of aldehydes on MOF-199 and UiO-66-NH_2_, the results of theoretical modeling also demonstrated the influence of already adsorbed species on further adsorption of other species. In case of MOF-199, the adsorption of small aldehydes provided rather large distortion of lattice (Table S2). Correspondingly, the adsorption of larger aldehyde molecules became energetically more favorable (than MOF-5) due to lattice stabilization by molecule-linker interactions. In case of UiO-66-NH_2_, the picture was more complicated despite some similarities in the adsorption patterns with MOF-5 and MOF-199 (Refer to Supplementary Table S3). This is due to greater flexibility of this MOF (at low mechanical loading) and also due probably to additional contribution from integrations of –NH_2_ groups of ligands with adsorbed molecules.

### Comparison of results using MOF and commercial sorbent and effect of MOF storage time

**Figure 3:**
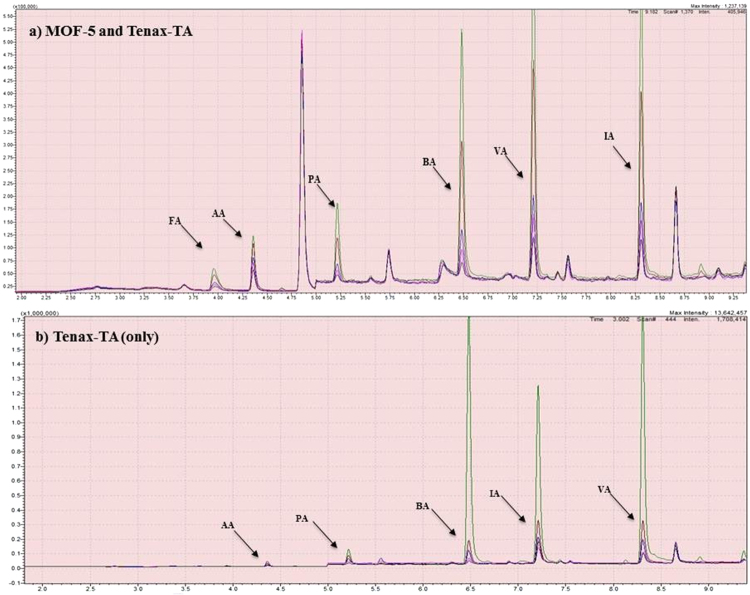
Chromatograms of a mixture of carbonyl compounds used as the L-WS (analytical volume = 1 µL) using MOF-5 + Tenax-TA as ST and CT sorbents. Colors represent different masses as follows: green = 95 to 98 ng, brown = 47 to 49 ng, blue = 23 to 24 ng, pink = 10 ng, black = 4.8 ng.

The results of analyzing CC mixtures using a MOF-5 + Tenax-TA-based TD-GC/MS system were compared with the results using the system with Tenax-TA only (Fig. 3 and Supplementary Table S4). Accordingly, detection of C2 to C5 aldehydes was achieved using both Tenax-TA (on its own) and the MOF-Tenax combination. However, FA (a C1 molecule) alone could only be detected with the multi-adsorbent-based system. FA was not detected using Tenax-TA on its own. Simultaneous detection of all aldehyde species with the multi-adsorbent system is likely a consequence of the partial adsorption of heavier molecules onto the commercial sorbent (Tenax-TA) lessening the competition (between FA and larger aldehyde species) for the available MOF pores (MOF-5). The effect of storage time of MOF was also investigated. The results using old and freshly prepared MOFs were comparable (Refer to Supplementary Table S4), although the use of fresh MOF resulted in slightly greater RF values (with larger RF values for larger molecules) than when older MOFs were used.

## Conclusions

In this work, the feasibility of a MOF-based multi-adsorbent technique was demonstrated successfully for the first time for the simultaneous analysis of aldehyde molecules containing 1 to 5 C atoms by TD-GC/MS. The multi-adsorbent-based approach offered higher sensitivity and better reproducibility than that built with a commercial sorbent without the requirement of complex derivatization of small aldehyde molecules. The performance of Zn-based MOF-5 was demonstrated to be superior both theoretically and experimentally to that of Cu-based MOF-199 and Zr-based MOF (of UiO-66 topology). Furthermore, the performance of MOF-5 system was not affected considerably by the storage of MOF. The result of the theoretical modeling analyses revealed that the structural and chemical characteristics of the MOF component of the binary sorbent material should have a direct effect on the efficacy of TD-GC/MS quantification of carbonyl mixtures. Nevertheless, there are still some difficulties associated with co-adsorption on the MOF, which may affect the uptake of lighter compounds. Further work should thus be directed to help resolve these issues, perhaps by establishing more reliable method based on the analysis of ambient air samples and by continuing efforts to develop more materials with advanced functionalities.

## Materials and Methods

### Chemicals and synthesis of the adsorbents

Reagents were commercially available products that were used without any further purification. Zinc nitrate hexahydrate (Zn(NO_3_)_2_·6H_2_O) (98%), terephthalic acid (1,4-benzenedicarboxylate, BDC) (98%), amino terephthalic acid (BDC-NH_2_), and trimethylamine (TEA) (≥99%) were procured from Sigma-Aldrich. Chloroform (>99.5%) was procured from Daejung, Korea. Dimethylformamide (DMF, 99.0%) and ethanol (99.5%) were obtained from Samchun Chemicals, Korea. Tenax-TA (35/60 mesh) was obtained from Markes International, UK.

MOF-5 was synthesized as follows: 25 mL of DMF were placed in a 100-mL beaker. While the DMF was stirred continuously at 1,000 rpm using a magnetic stir bar on a digital hotplate stirrer (DAIHAN Scientific, Korea), 0.3 g of terephthalic acid was added to prepare the linker solution. Similarly, 1.4 g of Zn(NO_3_)_2_·6H_2_O was added to 25 ml of DMF to make the metal solution. The linker solution was mixed with the metal solution and stirred constantly. Then, 2 mL of TEA was added drop-wise to the reaction mixture as a catalyst (*in situ* synthesis method) to reduce the reaction time for MOF-5 synthesis. The solution was then covered with aluminum foil and left for 2 hours at 25 °C. The precipitate in the solution was filtered using a glass microfiber filter (diameter 47 mm, Whatman^TM^, UK) under vacuum applied using a mini diaphragm vacuum pump (N86KT.18, KNF, UK) (pressure difference = 2 bar and filtering time = 5 min). The residue was washed two times with DMF to remove excess BDC. The DMF was exchanged with 40 ml chloroform and allowed to soak for 12 hours before it was exchanged with fresh chloroform. This process was repeated twice. The chloroform was filtered without letting the MOF dry, and the product was dried in a convection oven (CO-150, Hanyang Scientific Equipment Co., Ltd, Korea) for 8 hours at 90 °C.

MOF-199 was synthesized by dissolving 10 g of Cu (NO_3_)_2_∙2.5H_2_O and 5 g of H_3_BTC in 250 ml of solvent containing a 1:1:1 ratio of DMF, ethanol, and water. Later, the mixture was transferred to a tightly capped glass vessel and heated at 85 °C for 20 h in a forced convection oven (CO-150, Hanyang Scientific Equipment Co., Ltd, Korea). The resulting blue crystals were washed with 30 mL of DMF two times to remove the unbounded ligand materials. The resulting product was then immersed in DMF (30 mL) for 24 h to remove the undissolved impurities. The product was filtered using a glass fiber filter (diameter 47 mm, Whatman^TM^, UK) under vacuum using a mini diaphragm vacuum pump (N86KT, KNF, UK) (pressure difference was 2 bar, and filtering time was 10 minutes). Finally, the filtered product was kept in an oven at 170 °C for 24 h.

The synthesis of UiO-66-(NH_2_) was based on the protocol reported by Katz *et al*.^15^. Briefly, the metal solution was prepared by dissolving 125 mg of ZrCl_4_∙8H_2_O in 5 mL of DMF to which 1 mL of concentrated HCl was added. The resulting mixture was then subjected to ultra-sonication for about 20 min. The organic linker solution was prepared simultaneously by dissolving 134 mg of amino terephthalic acid (BDC-NH_2_) in 10 mL of DMF. The above metal and organic linker solutions were mixed and ultrasonicated for an additional 30 min to complete the reaction. The resulting solution produced a pale yellow solid that precipitated after heating at 80 °C for 12 h. These solids were filtered and then thoroughly washed two times with DMF (30 ml) and two times with ethanol (30 ml). The resulting product was allowed to soak in ethanol (10 mL) three times in three days and heated at 90 °C for 12 h. The final solid product was stored in a closed vial at room temperature under atmospheric conditions.

Finally, an empty quartz sorbent tube (ST) (length: 89 mm, OD: 6 mm, and ID: 4 mm, Top Trading Co., Korea) was packed with MOF and Tenax-TA (at a ratio of 1:1 by mass). The ST was first packed with Tenax-TA and then with MOF (refer to Supplementary Fig. S5 for details.) The two adsorbents were separated and held in place using quartz wool. The total mass of the sorbent was 60 mg. The ends of the quartz tube were plugged with quartz wool. The ST was thermally conditioned at 150 °C for 20 hours (purge gas = 99.999% nitrogen and purge flow rate = 200 mL min^−1^) to make sure no impurities remained adsorbed.

### Characterization of MOFs

The powder X-ray diffraction (PXRD) patterns of the MOFs activated at 150 °C for 2 hours were recorded with an HR-XRD diffractometer (Rigaku, Japan). Data were collected with an angular 2Ɵ range of 5–60°, a step size of 0.02°, and a scan speed of 4° min^−1^. The thermal stability of MOF-5 was determined by monitoring the loss of mass of MOF-5 as a function of temperature in a thermogravimetric analysis (TGA) experiment. TGA experiments were performed using a SDTQ600 Auto-DSCQ20 system (Eden Prairie, MN, USA). Samples were placed in alumina pans heated from 25 to 800 °C at a ramping rate of 10 °C min^−1^ while supplying ultrapure N_2_ at 100 ml min^−1^. Additionally, the morphological characteristics of the MOFs were studied using a NOVA nanoSEM-450 field emission scanning electron microscope (FE-SEM, Hillsboro, OR, USA) to obtain images of MOFs. The metal-ligand binding of pristine and used MOF was examined using Fourier transform infrared spectroscopy (FTIR) analysis. This analysis confirmed that the MOFs had been prepared correctly.

### Preparation of liquid working standards (L-WS)

Reagent grade chemicals (RGC) were purchased from Sigma-Aldrich (USA), and the FA solution and mixture of aldehydes were prepared. The liquid primary standard (LPS) was diluted with methanol for the preparation of working standards (L-WS) at five different concentrations (Supplementary Tables S5 and S6). The structure of MOF-5 can be altered by intrusion of water. However, the very low concentration of water impurities (<35 ng μL^−1^) in the L-WS made the extent of this degradation negligible.

### Instrumental (TD-GC/MS) system

All carbonyl samples in this study were analyzed using a GC (Shimadzu GC-2010, Japan) equipped with an MS (Shimadzu GCMS-QP2010 Ultra, Japan) and a TD (Unity II, Markes International Ltd, UK) front end. The cold trap (CT) in the TD unit was packed with MOF-5 and Tenax-TA at a ratio of 1:1 by volume. These components were separated by quartz wool (QW), and the unit had an inner diameter of 2 mm and a total (sorbent + QW) bed length was 7 cm (2.4 cm for both MOF-5 and Tenax-TA) (refer to Supplementary Fig. S5). The analytes (i.e., individual or mixtures of aldehydes) loaded onto the ST were subjected to thermal desorption to transfer them for separation using a CP-Wax column (diameter: 0.25 mm, length: 60 m, and thickness: 0.25 µm). Figure 1 depicts the method of replacement of the cold (quartz) trap in the TD unit interfaced with the GC/MS system along with those for sorbent tube (ST) sampling and injection of L-WS into the ST. This study comprised of a total of seven experimental stages (E1 to E7) as the basic experimental scheme of each stage is outlined in Table 2. Final detection of all aldehydes was performed using the MS detection system. FA was quantified in selected ion monitoring (SIM) mode at 29, 30, and 44 m/z, and the other aldehydes were quantified in the total ion chromatogram (TIC) mode at 35 to 600 m/z (Table 3).

### Density functional theory (DFT) calculations

The interaction mechanism between the MOF and the guest analytes was studied by the DFT calculations. For this purpose, the pseudo-potential code SIESTA model was applied^33^. All the calculations were carried out using the generalized gradient approximation (GGA-PBE) with spin-polarization^34^ and the +*vdw* correction^35^ required for the description of weak interactions. A full optimization of atomic positions was performed. In the course of the optimization, the ion cores were described by norm-conserving non-relativistic pseudo-potentials^36^ with cut-off radii of 2.52, 2.37, 2.08, 1.15, 1.14, 1.45, and 1.25 au for Zr, Zn, Cu, O, C, N, and H, respectively. Moreover, the wave functions were expanded with a double-ζ plus polarization basis of localized orbitals for all species (excluding hydrogen), while a double-ζ basis for hydrogen. Optimization of the force and total energy was done with an accuracy of 0.04 eV/Å and 1 meV, respectively. These calculations were performed with an energy mesh cut-off of 300 Ry and a k-point mesh of 4 × 4 × 4 in the Mokhorst-Pack scheme. Electronic structure of all molecules was calculated for a single molecule located in the empty cubic box with sides of 20 Å. This procedure was considered to reflect realistic atomic structures of MOF-5 and MOF-199 based on the Cambridge Database (CCDC) and of UiO-66-NH_2_ taken from Valenzano *et al*.^37^. The DFT-based 0 K adsorption enthalpy (ΔH_ab_) was calculated by

1 $${{\rm{\Delta }}H}_{{\rm{ab}}}=({{\rm{E}}}_{{\rm{host}}+{\rm{guest}}})-({{\rm{E}}}_{{\rm{host}}}+{{\rm{E}}}_{{\rm{guest}}})$$

where E_host+guest_ is the total energy of the MOF and the guest analyte, E_host_ is the total energy of the pristine MOF, and E_guest_ is the energy of the guest molecule in the gas phase.

### Data availability

The datasets generated from this study are included in this published article and in Supplementary information.

## Electronic supplementary material

Supplementary information
