# Supplementary material for: Metal-organic framework and Tenax-TA as optimal sorbent mixture for concurrent GC-MS analysis of C1 to C5 carbonyl compounds
Source: Sci Rep. 2018 Mar 22;8:5033. doi: 10.1038/s41598-018-23391-6 (PMC5864741; doi:10.1038/s41598-018-23391-6)
Supplement: Supplementary file 1 — Supplementary information [file 41598_2018_23391_MOESM1_ESM.doc]

**­Supporting Material**

**Metal-organic framework and Tenax-TA as optimal sorbent mixture for concurrent GC-MS analysis of C1 to C5 carbonyl compounds**

Tanushree Dutta^a^, Ki-Hyun Kim^a^,*, Richard J. C. Brown^b^, Yong-Hyun Kim^c^, Danil Boukhvalov^d^

^a^Department of Civil and Environmental Engineering, Hanyang University, 222 Wangsimni-Ro, Seoul 04763, Korea

^b^Department of Chemical, Medical and Environmental Science, National Physical Laboratory, Teddington, TW11 0LW, UK

^c^Jeonbuk Department of Inhalation Research, Korea Institute of Toxicology, Jeongeup 56212, Republic of Korea

^d^Department of Chemistry, Hanyang University, 222 Wangsimni-Ro, Seoul 04763, Korea

Table S1. Enthalpies of adsorption (in eV/molecule) of the second molecules in the MOF-5 with an already adsorbed molecule of the first type, and the ratio of the volume after adsorption and pristine MOF-5. The combinations with the most significant deviations of the enthalpy from the values for same concentration in the case of adsorption of same species are indicated by red.

| First/Second | FA | AA | PA | BA | VA | IA |
| --- | --- | --- | --- | --- | --- | --- |
| FA | -1.251  100.0 | -0.544  99.3 | -1.468  99.9 | -1.376  99.8 | -1.113  99.9 | -1.271  99.8 |
| AA | -0.302  99.3 | -1.477  99.9 | -1.451  99.8 | -1.037  99.7 | -1.174  99.7 | -1.366  99.8 |
| PA | -1.196  99.9 | -1.421  99.8 | -1.514  99.9 | -1.467  100.1 | -1.507  100.0 | -1.643  99.9 |
| BA | -1.093  99.8 | -0.996  99.7 | -1.458  100.1 | -1.530  100.1 | -1.541  100.0 | -1.6775  100.0 |
| VA | -0.823  99.9 | -1.136  99.7 | -1.489  100.0 | -1.534  100.0 | -1.524  99.9 | -1.635  100.0 |
| IA | -0.824  99.8 | -1.161  99.8 | -1.468  99.9 | -1.514  100.0 | -1.478  100.0 | -1.678  100.0 |

Table S2. Enthalpies of adsorption (in eV/molecule) of the second molecules in the MOF-199 with an already adsorbed molecule of the first type, and the ratio of the volume after adsorption and pristine MOF-199. The combinations with the most significant deviations of the enthalpy from the values for same concentration in the case of adsorption of same species are indicated by red if adsorption of the second species is blocked and green if became more favorable.

| First/Second | FA | AA | PA | BA | VA | IA |
| --- | --- | --- | --- | --- | --- | --- |
| FA | -0.102  97.3 | -0.203  97.0 | -0.147  96.9 | -0.189  97.1 | -0.181  97.2 | -0.238  97.0 |
| AA | -0.153  97.0 | -0.096  97.1 | -0.154  96.9 | -0.969  96.7 | -0.219  97.1 | -0.226  97.2 |
| PA | +0.103  96.9 | +0.045  97.1 | -0.118  98.1 | -0.135  98.3 | -0.158  98.3 | -0.167  98.2 |
| BA | +0.115  97.1 | +0.158  96.7 | -0.080  98.3 | -0.108  98.4 | -0.101  98.4 | -0.131  98.2 |
| VA | +0.131  97.2 | +0.043  97.1 | -0.097  98.3 | -0.095  98.4 | -0.154  98.8 | -0.123  98.6 |
| IA | +0.134  97.0 | +0.097  97.2 | -0.044  98.2 | -0.063  98.2 | -0.061  98.6 | -0.146  98.8 |

Table S3. Enthalpies of adsorption (in eV/molecule) of the second molecules in the UiO-66-NH_2_ with an already adsorbed molecule of the first type, and the ratio of the volume after adsorption and pristine UiO-66-NH_2_. The combinations with the most significant deviations of the enthalpy from the values for same concentration in the case of adsorption the same species are indicated by red if adsorption of the second species is blocked and green if became more favorable.

| First/Second | FA | AA | PA | BA | VA | IA |
| --- | --- | --- | --- | --- | --- | --- |
| FA | -0.040  107.4 | -0.002  105.8 | -0.091  107.3 | -0.141  107.5 | -0.149  107.6 | -0.128  107.1 |
| AA | +0.013  105.8 | -0.027  106.5 | -0.055  106.1 | -0.011  107.0 | -0.041  105.6 | +0.052  105.5 |
| PA | -0.049  107.3 | -0.028  106.1 | -0.125  106.9 | -0.204  107.2 | -0.182  106.8 | -0.163  107.0 |
| BA | -0.026  107.5 | -0.089  107.0 | -0.132  107.2 | -0.160  107.2 | -0.105  106.9 | -0.079  107.0 |
| VA | -0.039  107.6 | +0.054  105.6 | -0.114  106.8 | -0.110  106.9 | -0.102  106.7 | +0.059  107.2 |
| IA | -0.013  107.1 | +0.151  105.5 | -0.090  107.0 | -0.078  107.0 | -0.054  107.2 | +0.007  107.6 |

Table S4. Comparison of results from old (storage time=6 months) and freshly prepared (storage time<1 month) MOF-5 and Tenax-TA under optimum conditions.

|  |  | Adsorption temperature=-25°C | | | | | |
| --- | --- | --- | --- | --- | --- | --- | --- |
|  |  | Tenax TA | | TenaxTA+MOF-5 | | TenaxTA+MOF-5 | |
|  |  |  |  | **Storage time=6 months** | | **Storage time< 1 month** | |
| Order | Compound | RF value | R^2^ | RF value | R^2^ | RF value | R^2^ |
| 1 | FA | - | - | 1,952 | 0.9971 | 2,762 | 0.8086 |
| 2 | AA | 705 | 0.9945 | 5,267 | 0.7791 | - | - |
| 3 | PA | 3,312 | 0.9863 | 4,388 | 0.9900 | 3,926 | 0.9858 |
| 4 | BA | 8,465 | 0.9973 | 12,722 | 0.9973 | 10,723 | 0.9851 |
| 5 | IA | 15,320 | 0.8417 | 17,738 | 0.9973 | 14,190 | 0.9768 |
| 6 | VA | 15,549 | 0.9528 | 24,362 | 0.9969 | 13,097 | 0.9768 |

| Table S5. Preparation of liquid working standards (L-WS) | | | | |  |  |  |  |  |
| --- | --- | --- | --- | --- | --- | --- | --- | --- | --- |
|  |  | *Reagent grade chemical (RGC)* | | |  | *Primary standard (PS)* | | |  |
| Order | Compounds | Density | Concentration | |  | Dilution of RGC | |  | Concentration |
|  |  | (g mL^-1^) | (wt,%) | (ng μL^-1^) |  | (mg) | (μL) |  | (ng μL^-1^) |
| 1 | FA | 0.9046 | 37 | 334,702 |  | 18.1 | 20.0 |  | 3,719 |
| 2 | H_2_O | 1.0 | 12.5 | 125,000 |  |  |  |  | 1,389 |
| 3 | MeOH | 0.792 | 99.8 | 790,416 |  | 1,410 | 1,780 |  | 781,633 |
|  |  |  |  |  | Total: | 1,428 | 1,800 |  |  |
|  |  |  |  |  |  |  |  |  |  |
| *Liquid working standard (L-WS)* | | | |  |  |  |  |  |  |
| Order | Dilution volume (μL) | | Dilution fraction | Concentration (ng μL^-1^) |  |  |  |  |  |
|  | PS | MeOH (RGC) |  | FA |  |  |  |  |  |
| 1 | 2 | 1,798 | 0.001 | **4.13** |  |  |  |  |  |
| 2 | 4 | 1,796 | 0.002 | **8.27** |  |  |  |  |  |
| 3 | 10 | 1,790 | 0.006 | **20.67** |  |  |  |  |  |
| 4 | 20 | 1,780 | 0.011 | **41.34** |  |  |  |  |  |
| 5 | 40 | 1,760 | 0.022 | **82.68** |  |  |  |  |  |

Table S6. Preparation of liquid working standards (L-WSs) of mixtures of carbonyls

| *Reagent grade chemical (RGC)* | | |  |  | *Primary standard (PS): Mixing of RGC in 2 ml vial* | | | | |
| --- | --- | --- | --- | --- | --- | --- | --- | --- | --- |
| Order | Compound name | Concentration (%) | Density (g mL^-1^) |  | RGC Volume (μL) | Dilution fraction | Concentration | |  |
|  |  |  |  |  |  |  | (%) | (ng μL^-1^) |  |
| 1 | **FA** | 37 | 0.9046 |  | 460 | 0.256 | 9.46 | 85535 |  |
|  | **H_2_O** | 12.5 | 1.0 |  |  |  | 3.19 | 31944 |  |
| 2 | **AA** | 99.5 | 0.785 |  | 200 | 0.111 | 11.06 | 86,786 |  |
| 3 | **PA** | 97.0 | 0.798 |  | 200 | 0.111 | 10.78 | 86,007 |  |
| 4 | **BA** | 99.0 | 0.805 |  | 200 | 0.111 | 11.00 | 88,550 |  |
| 5 | **IA** | 97.0 | 0.797 |  | 200 | 0.111 | 10.78 | 85,899 |  |
| 6 | **VA** | 97.0 | 0.810 |  | 200 | 0.111 | 10.78 | 87,300 |  |
| 7 | **MeOH** | 99.8 | 0.792 |  | 340 | - | - | - |  |
|  |  |  |  |  |  |  |  |  |  |
| *1st working standard: Dilution of PS in 20 ml vial* | | | | *Final WS: Dilution of 1st WS in 2 ml vial* | | | |  |  |
| PS volume (μL) | Dilution fraction | Concentration |  | 1st WS volume (µL): | 10 | 20 | 50 | 100 | 200 |
|  |  | (ng μL^-1^) |  | MeOH (RGC): | 1,790 | 1,780 | 1,750 | 1,700 | 1,600 |
| 180 | 0.01 | 855.3 |  | **FA** | 4.8 | 9.5 | 23.8 | 47.5 | 95.0 |
|  |  | 319.4 |  | **H_2_O** | 1.8 | 3.5 | 8.9 | 17.7 | 35.5 |
|  |  | 867.9 |  | **AA** | 4.8 | 9.6 | 24.1 | 48.2 | 96.4 |
|  |  | 860.1 |  | **PA** | 4.8 | 9.6 | 23.9 | 47.8 | 95.6 |
|  |  | 885.5 |  | **BA** | 4.9 | 9.8 | 24.6 | 49.2 | 98.4 |
|  |  | 859.0 |  | **IA** | 4.8 | 9.5 | 23.9 | 47.7 | 95.4 |
|  |  | 873.0 |  | **VA** | 4.9 | 9.7 | 24.3 | 48.5 | 97.0 |
| 17820 |  |  |  |  |  |  |  |  |  |


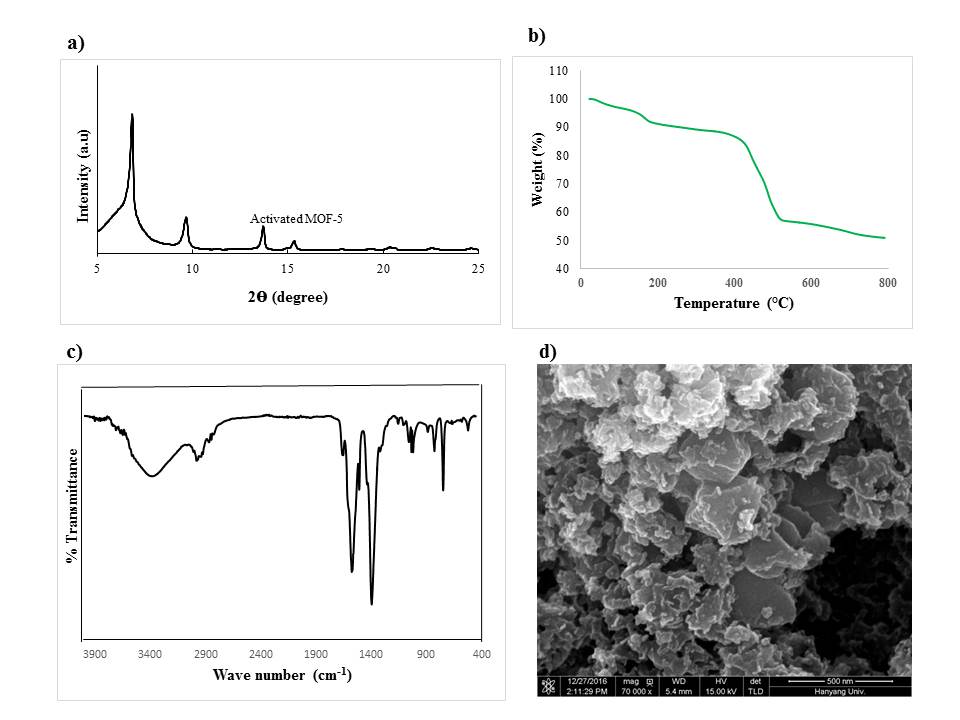


Figure S1. Results of characterization of MOF-5 using a) PXRD, b) TGA, c) FTIR, and d) SEM.


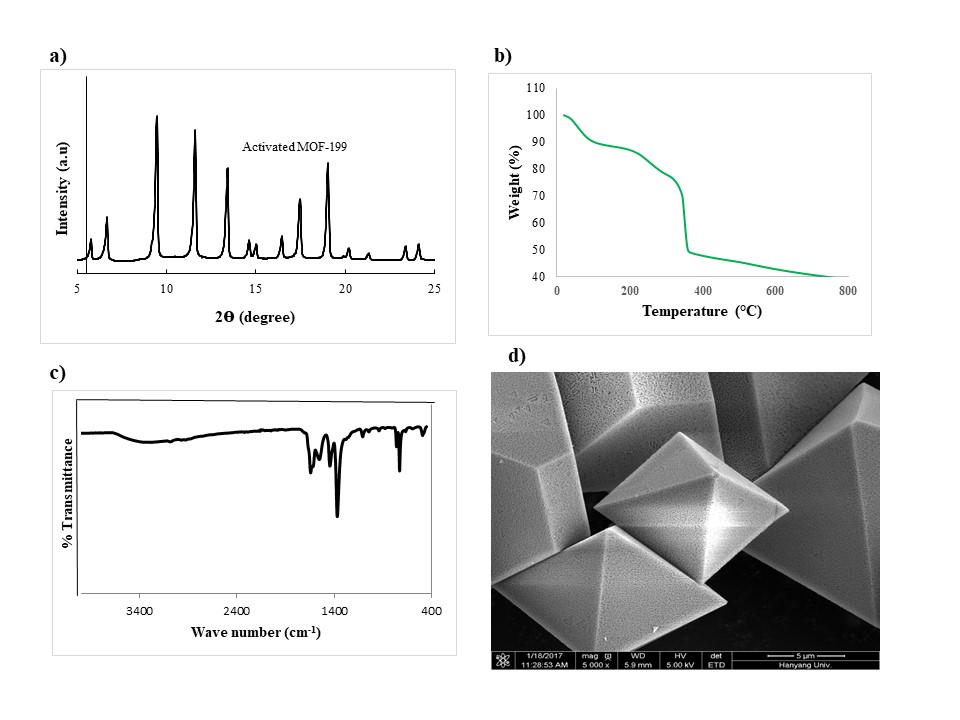


Figure S2. Results of the characterization of MOF-199 using a) PXRD, b) TGA, c) FTIR, and d) SEM.


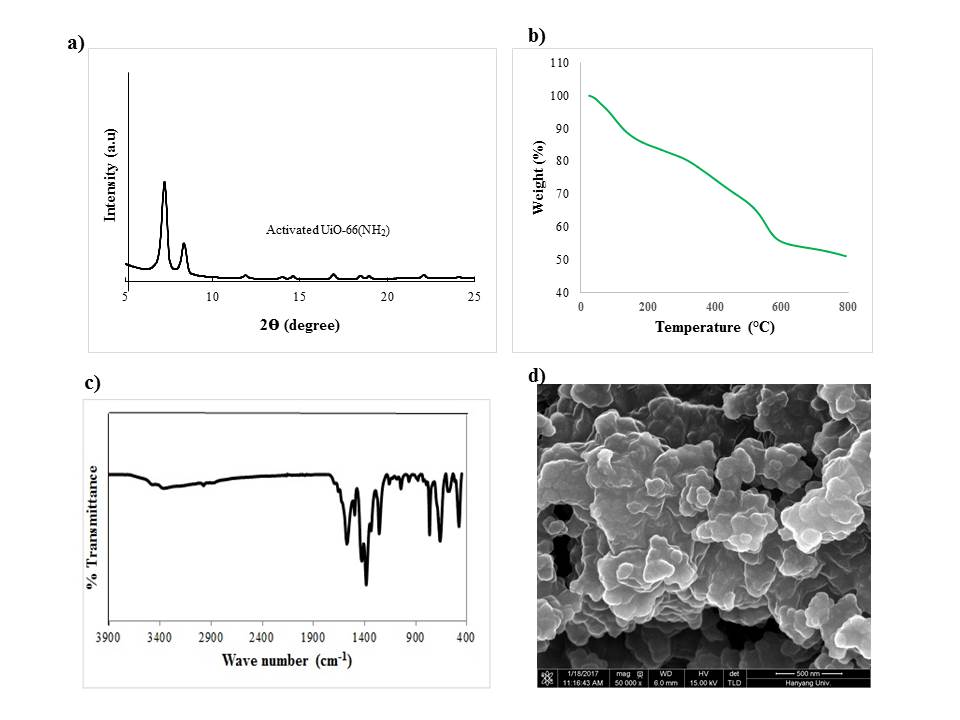


Figure S3. Results of the characterization of UiO-66(NH_2_) using a) PXRD, b) TGA, c) FTIR, and d) SEM.


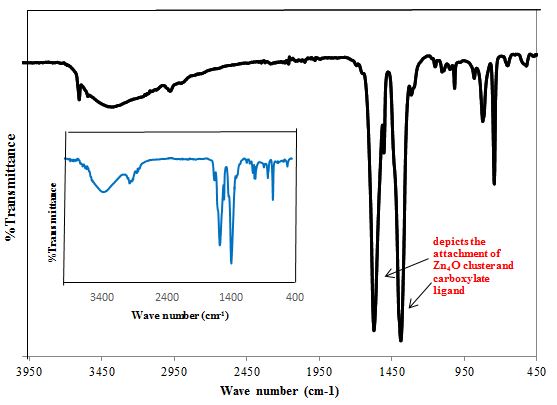


Figure S4. FTIR characterization showing the comparison between the pristine MOF-5 (blue) and MOF-5 (from cold trap) (black) after the desorption of adsorbed carbonyl molecules.


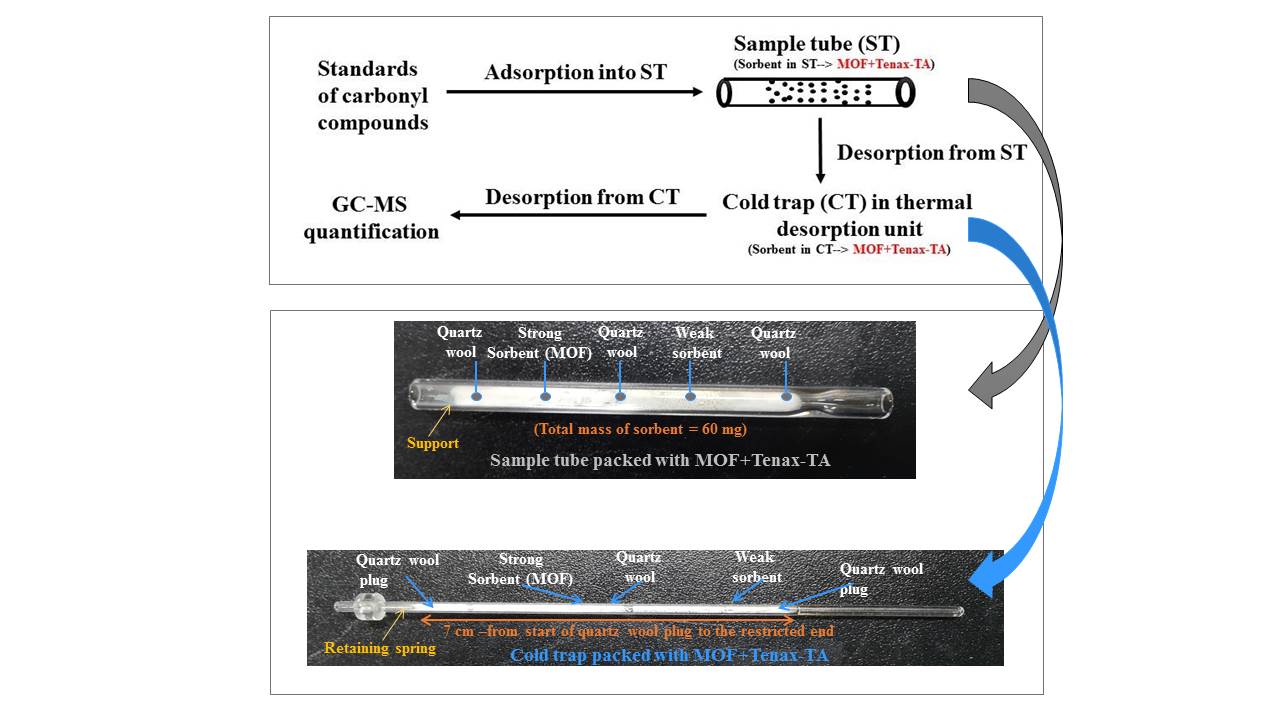


Figure S5. Schematic of sampling and desorption of carbonyls employing sorbent tube (ST) and a cold trap (CT) packed with MOF and Tenax-TA.


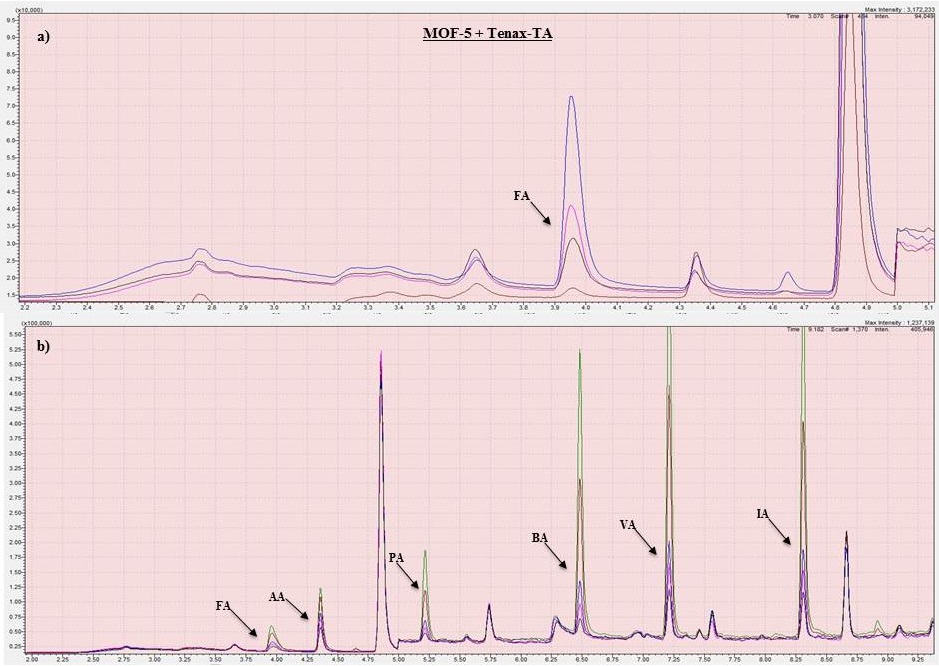


Figure S6. Chromatograms showing the retention times of a) FA (single species) and b) carbonyl compounds (multiple species including FA) obtained for the L-WS (analytical volume= 1 µL) using MOF-5+Tenax-TA as a ST and a CT sorbent. Colors represent different masses as follows: green= 95 to 98 ng, brown=47 to 49 ng, blue=23 to 24 ng, pink=10 ng, black=4.8 ng.


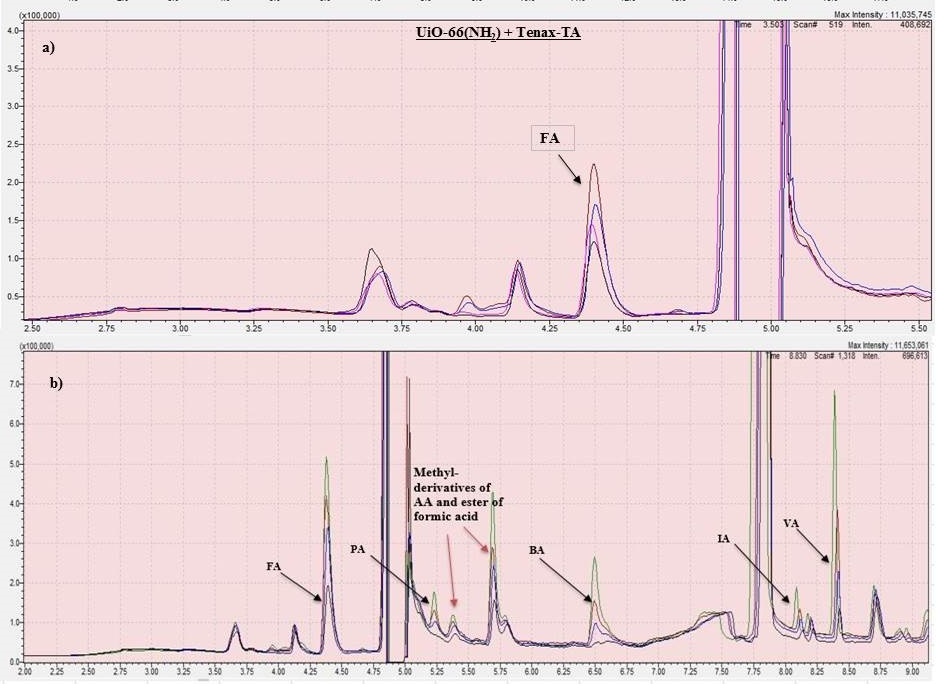


Figure S7. Chromatograms of a) FA (single species) and b) carbonyl compounds (multiple species including FA) obtained for the L-WS (analytical volume= 1 µL) using UiO-66(amine derivative) +Tenax-TA as a ST and a CT sorbent. Colors represent different masses as follows: green= 82 ng, brown=41 ng, blue=21 ng, pink=8 ng, black=4 ng.


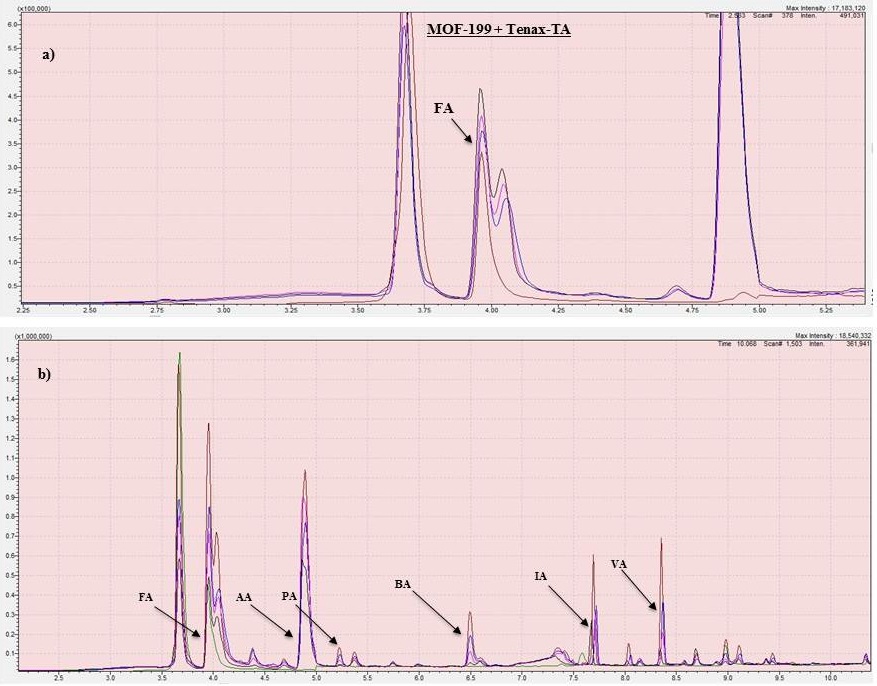


Figure S8. Chromatograms of a) FA (single species) and b) carbonyl compounds (multiple species including FA) obtained for the L-WS (analytical volume= 1 µL) using MOF-199 +Tenax-TA as a ST and a CT sorbent. Colors represent different masses as follows: green= 82 ng, brown=41 ng, blue=21 ng, pink=8 ng, black=4 ng.
